# Severe hyperprolactinemia secondary to primary hypothyroidism with normal pituitary imaging

**DOI:** 10.1210/jcemcr/luag069

**Published:** 2026-04-21

**Authors:** Alpana Meena

**Affiliations:** LNCT & JK Hospital, Bhopal, Madhya Pradesh 462042, India

**Keywords:** hyperprolactinemia, primary hypothyroidism, pituitary imaging, TRH-mediated prolactin secretion, menstrual irregularity

## Abstract

Hyperprolactinemia is often attributed to pituitary adenomas; however, primary hypothyroidism is an important and often overlooked secondary cause. We report the case of a 36-year-old woman who presented with giddiness, fatigue, menstrual irregularity, and markedly elevated serum prolactin levels 339 ng/mL (SI: 339 µg/L); reference range, 5-25 ng/mL (SI: 5-25 µg/L) with normal pituitary imaging. Biochemical evaluation revealed severe primary hypothyroidism with significantly elevated thyroid-stimulating hormone levels. Treatment with levothyroxine alone resulted in clinical and biochemical improvement, confirming the diagnosis of hypothyroidism-induced hyperprolactinemia. This case highlights the importance of evaluating thyroid function in patients with hyperprolactinemia to avoid misdiagnosis and unnecessary treatment.

## Introduction

Hyperprolactinemia is a common endocrine disorder, frequently associated with pituitary adenomas, medications, or physiological states such as pregnancy and lactation. Primary hypothyroidism is a recognized but underappreciated cause of hyperprolactinemia, mediated by increased thyrotropin-releasing hormone (TRH) stimulation of lactotroph cells in the anterior pituitary [1, 2]. While prolactin elevations in hypothyroidism are typically modest, marked elevations may occur and mimic prolactin-secreting pituitary adenomas, leading to unnecessary imaging and treatment [3].

We report a case of severe hyperprolactinemia in a patient with primary hypothyroidism and normal pituitary imaging, emphasizing the diagnostic importance of a systematic endocrine evaluation.

## Case presentation

A 36-year-old woman presented with one-week history of giddiness, associated with headache and generalized fatigue. She also reported bilateral lower limb swelling, irregular menstrual cycles, hair loss and progressive reduction of axillary and pubic hair over the preceding several months. There was no history of galactorrhea, visual disturbances, seizures, or use of medications known to elevate prolactin levels. There was no history suggestive of chronic systemic illness.

On examination, the patient appeared fatigued. She had non-pitting pedal edema. Vital signs were stable. There were no features suggestive of acromegaly or Cushing syndrome. Neurological examination was unremarkable, and visual fields were normal on confrontation testing.

## Diagnostic assessment

Laboratory evaluation revealed hemoglobin of 10.4 g/dL (SI: 104 g/L) (reference range, 12.0-15.5 g/dL [SI: 120-155 g/L]) with a total leukocyte count of 8400/mm^3^ (SI: 8.4 × 10^9^/L) (reference range, 4000–11 000/mm^3^ [SI: 4.0-11.0 × 10^9^/L]). Fasting blood glucose was 88 mg/dL (SI: 4.9 mmol/L) (reference range, 70-99 mg/dL [SI: 3.9-5.5 mmol/L]).

Thyroid function tests showed severe primary hypothyroidism with a thyroid-stimulating hormone (TSH) level of 146 mIU/L (reference range, 0.4-4.5 mIU/L [SI: 0.4-4.5 mIU/L]) and low total triiodothyronine (T3) 0.35 ng/mL (SI: 0.54 nmol/L) (reference range, 0.8-2.0 ng/mL [SI: 1.23-3.08 nmol/L]) and total thyroxine (T4) 4.1 µg/dL (SI: 52.8 nmol/L) (reference range, 5.0-12.0 µg/dL [SI: 64.4-154.4 nmol/L]). Serum prolactin was markedly elevated at 339 ng/mL (SI: 339 µg/L) (reference range, 5-25 ng/mL [SI: 5-25 µg/L]). Anti-thyroid peroxidase antibodies were not measured.

Gonadotropin evaluation demonstrated low luteinizing hormone (LH) of 0.8 mIU/mL (SI: 0.8 IU/L) (reference range, 1.9-12.5 mIU/mL [SI: 1.9-12.5 IU/L]) and follicle-stimulating hormone (FSH) of 2.49 mIU/mL (SI: 2.49 IU/L) (reference range, 2.5-10.2 mIU/mL [SI: 2.5-10.2 IU/L]), consistent with hypogonadotropic hypogonadism in the setting of hyperprolactinemia.

Morning serum cortisol was elevated at 54.1 µg/dL (SI: 1493 nmol/L) (reference range, 5-25 µg/dL [SI: 138-690 nmol/L]), but the patient had no clinical features of Cushing syndrome. This was interpreted as a stress-related response. Repeat morning cortisol subsequently decreased to 22.9 µg/dL (SI: 632 nmol/L). There were no clinical features to suggest adrenal insufficiency.

Given the degree of hyperprolactinemia, pituitary magnetic resonance imaging was performed and demonstrated a normal pituitary size and morphology, with no evidence of adenoma or pituitary hyperplasia. The overall findings supported hyperprolactinemia secondary to severe primary hypothyroidism.

## Treatment

The patient was initiated on levothyroxine replacement therapy at an appropriate weight-adjusted dose. Dopamine agonist therapy was withheld.

**Figure 1:**
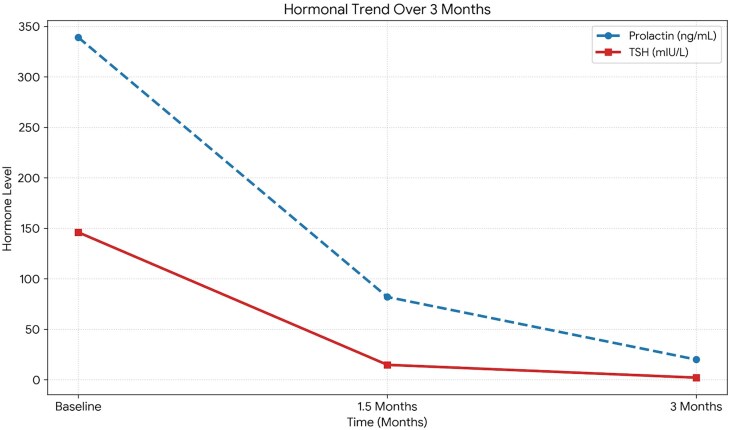
Hormonal timeline following levothyroxine therapy. Severe primary hypothyroidism at presentation (TSH 146 mIU/L) was associated with marked hyperprolactinemia (339 ng/mL). Following initiation of levothyroxine therapy, progressive normalization of thyroid function was observed with a parallel decline in serum prolactin, culminating in complete biochemical resolution at 3 months (TSH 2.11 mIU/L; prolactin 20 ng/mL), confirming hypothyroidism-induced hyperprolactinemia.

Serial biochemical monitoring demonstrated progressive normalization of thyroid function with concomitant resolution of hyperprolactinemia (Fig. 1).

## Outcome and follow-up

At 3-month follow-up, the patient reported complete resolution of giddiness and fatigue, with improvement in menstrual regularity. Biochemical reassessment demonstrated normalization of thyroid function with a TSH of 2.11 mIU/L and a corresponding normalization of serum prolactin to 20 ng/mL. (Table 1). Despite normalized TSH, total T3 remained below reference range, which may reflect assay variability or non-thyroidal influences; clinically she was euthyroid. These findings confirmed hypothyroidism-induced hyperprolactinemia, with complete biochemical resolution following levothyroxine therapy alone.

**Table 1:** Hormonal profile at presentation and at 3-month follow-up after levothyroxine therapy

Parameter	At presentation	3-month follow-up	Reference range
Serum Prolactin	339 ng/mL (SI: 339 µg/L)	20 ng/mL (SI: 20 µg/L)	5-25 ng/mL (SI: 5.0-25.0 µg/L)
TSH	146 mIU/L (SI: 146 mIU/L)	2.11 mIU/L (SI: 2.11 mIU/L)	0.4-4.5 mIU/L (SI: 0.4-4.5 mIU/L)
T4 (total)	4.1 µg/dL (SI: 52.8 nmol/L)	10.9 µg/dL (SI: 140.3 nmol/L)	5.0-12.0 µg/dL (SI: 64.4-154.4 nmol/L)
T3 (total)	0.35 ng/mL (SI: 0.54 nmol/L)	0.51 ng/mL (SI: 0.78 nmol/L)	0.8-2.0 ng/mL (SI: 1.23-3.08 nmol/L)
Cortisol (8AM)	54.1 µg/dL (SI: 1493 nmol/L)	22.9 µg/dL (SI: 632 nmol/L)	5-25 µg/dL (SI: 138.0-690.0 nmol/L)
LH	0.8 mIU/mL (SI: 0.8 IU/L)	Not repeated	1.9-12.5 mIU/mL (SI: 1.9-12.5 IU/L)
FSH	2.49 mIU/mL (SI: 2.49 IU/L)	Not repeated	2.5-10.2 mIU/mL (SI: 2.5-10.2 IU/L)

Anti-thyroid peroxidase antibodies were not measured. LH and FSH were not repeated at follow-up.

Abbreviations: FSH, follicle-stimulating hormone; LH, luteinizing hormone; T3, triiodothyronine; T4, thyroxine; TSH, thyroid-stimulating hormone.

## Discussion

Markedly elevated TSH level (146 mIU/L) together with hypogonadotropic hypogonadism and severe hyperprolactinemia strongly supports TRH-mediated lactotroph stimulation as the underlying mechanism. This case illustrates an important diagnostic pitfall in the evaluation of hyperprolactinemia. In the Endocrine Society clinical practice guideline, prolactin concentrations >250 µg/L (ng/mL) are highly predictive of prolactinoma, with approximately 99% of such cases attributable to prolactin-secreting adenomas [2]. Accordingly, the degree of prolactin elevation in this patient would typically prompt strong suspicion for prolactinoma and early initiation of dopamine agonist therapy.

In primary hypothyroidism, elevated TRH levels stimulate both thyrotroph and lactotroph cells, leading to increased secretion of TSH and prolactin [1]. Chronic untreated hypothyroidism may also result in pituitary hyperplasia, which can mimic macroadenoma on imaging and complicate diagnostic interpretation [4]. However, pituitary enlargement is not universal, and TRH-mediated hyperprolactinemia may occur despite normal pituitary morphology, reflecting a predominantly functional rather than structural pituitary response. Observational cohorts have reported that hyperprolactinemia is more frequent in overt hypothyroidism than in subclinical hypothyroidism and decreases significantly after restoration of euthyroidism with levothyroxine therapy [5]. Although prolactin elevations in hypothyroidism are typically mild to moderate, values in the range observed here remain uncommon. Consistent with this rarity, a published case report described primary hypothyroidism associated with an “exceptionally high” prolactin level of 323 ng/mL, reinforcing that severe elevations can occur in the absence of a prolactinoma [6].

Our patient had marked hyperprolactinemia with normal pituitary imaging, underscoring that even severe prolactin elevations do not exclude secondary etiologies. Early recognition and treatment prevented unnecessary pharmacological therapy and additional investigations. Complete normalization of prolactin levels by 3 months with thyroxine replacement alone reinforces guideline recommendations to exclude hypothyroidism before initiating dopamine agonist therapy [2]. This case adds to the existing literature by demonstrating that severe primary hypothyroidism may present with prolactin concentrations typically considered diagnostic of prolactinoma, yet remain fully reversible with thyroid hormone replacement alone.

## Learning points

- Primary hypothyroidism is an important and reversible cause of hyperprolactinemia.
- Severe prolactin elevation does not always indicate a prolactinoma.
- Thyroid function testing should be performed in all patients presenting with hyperprolactinemia.
- Correction of hypothyroidism alone may normalize prolactin levels and restore gonadal function.

## Data Availability

Original data generated and analyzed during this study are included in this published article.

## Abbreviations

FSH: follicle-stimulating hormone
LH: luteinizing hormone
T3: triiodothyronine
T4: thyroxine
TSH: thyroid-stimulating hormone
